# Supplementary figures and images for: Perfect mimicry between Heliconius butterflies is constrained by genetics and development
Source: Proc Biol Sci. 2020 Jul 22;287(1931):20201267. doi: 10.1098/rspb.2020.1267 (PMC7423669; doi:10.1098/rspb.2020.1267)

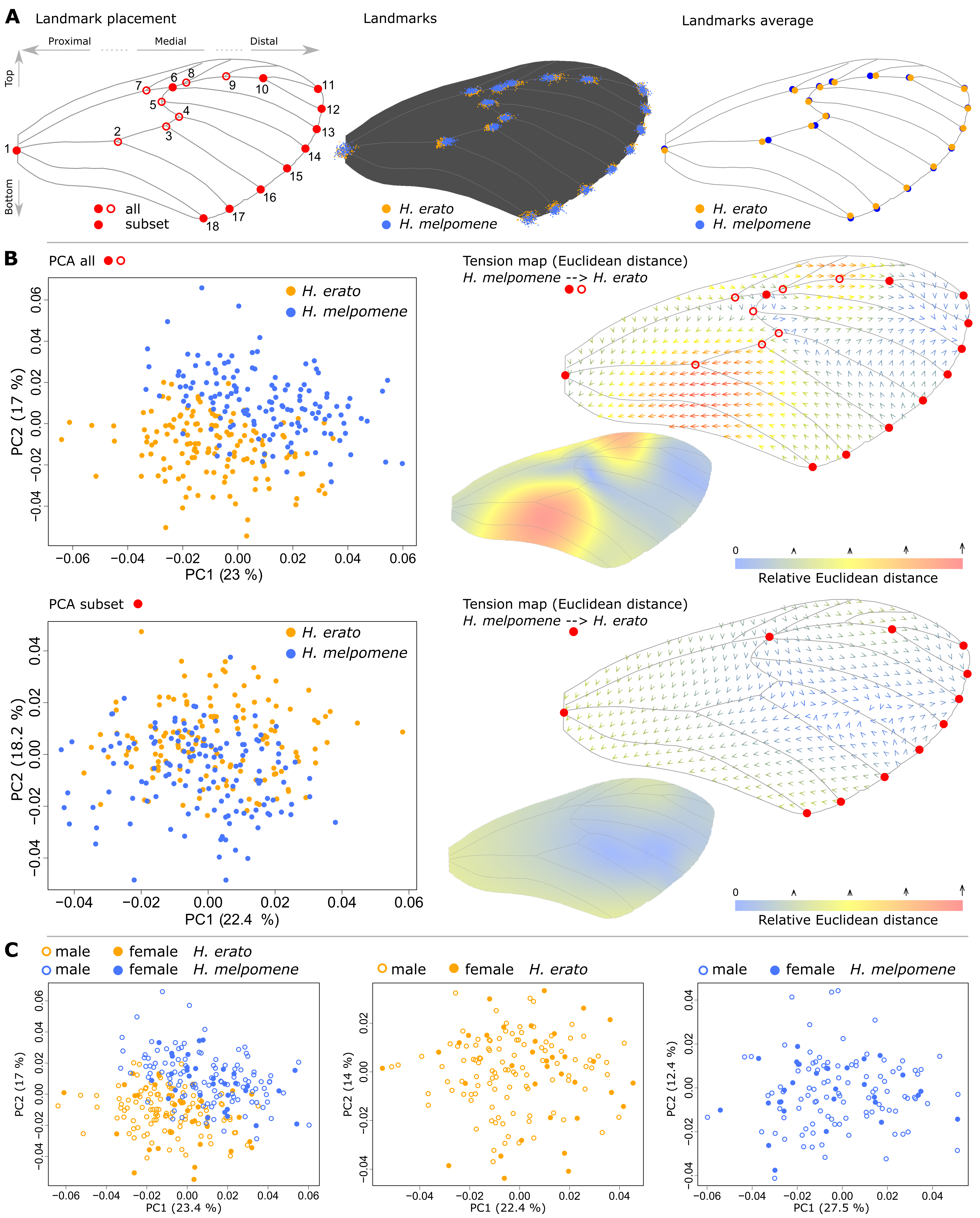

Supplement: Figure_S1.png [file rspb20201267supp1.png]

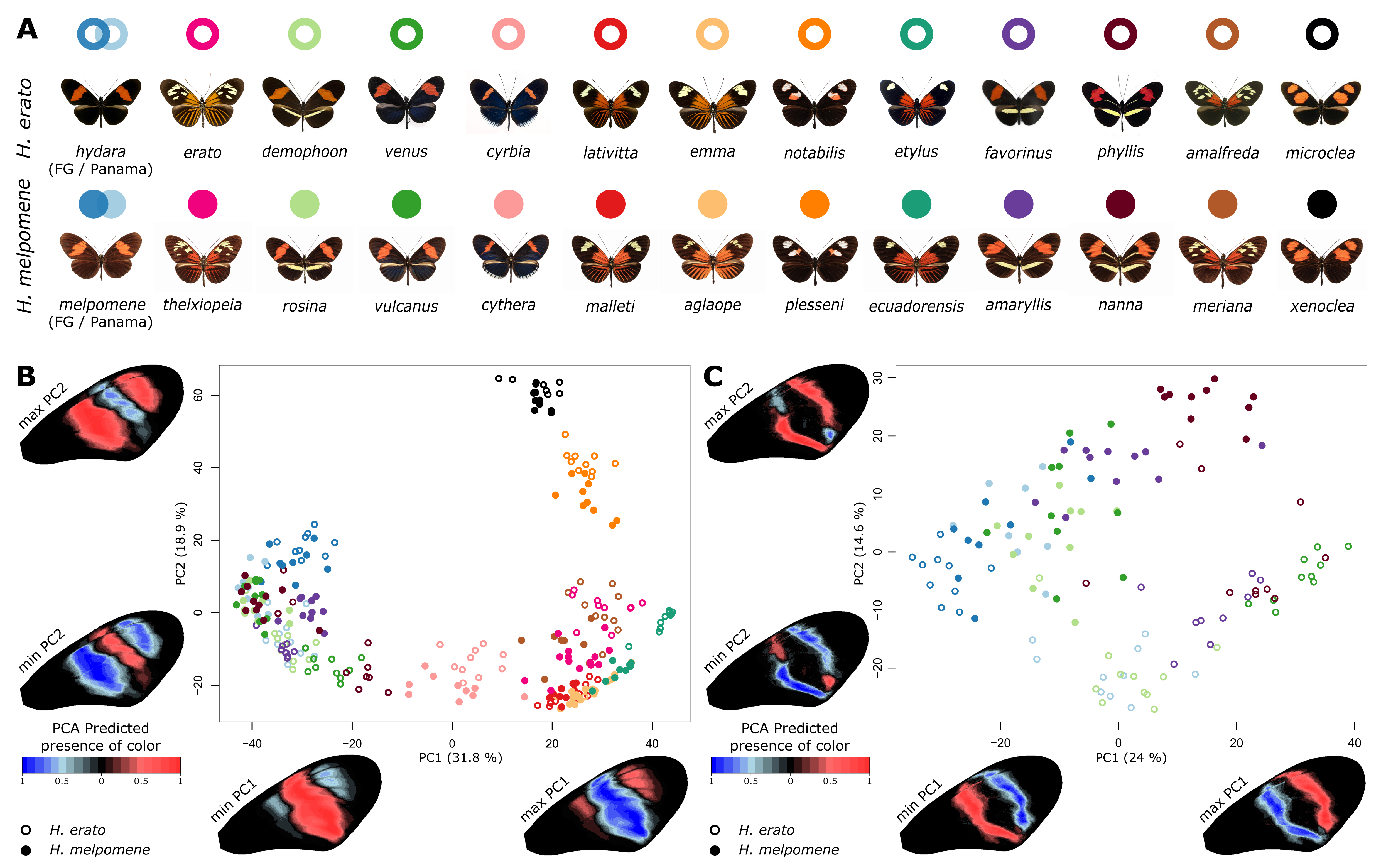

Supplement: Figure_S2.png [file rspb20201267supp2.png]

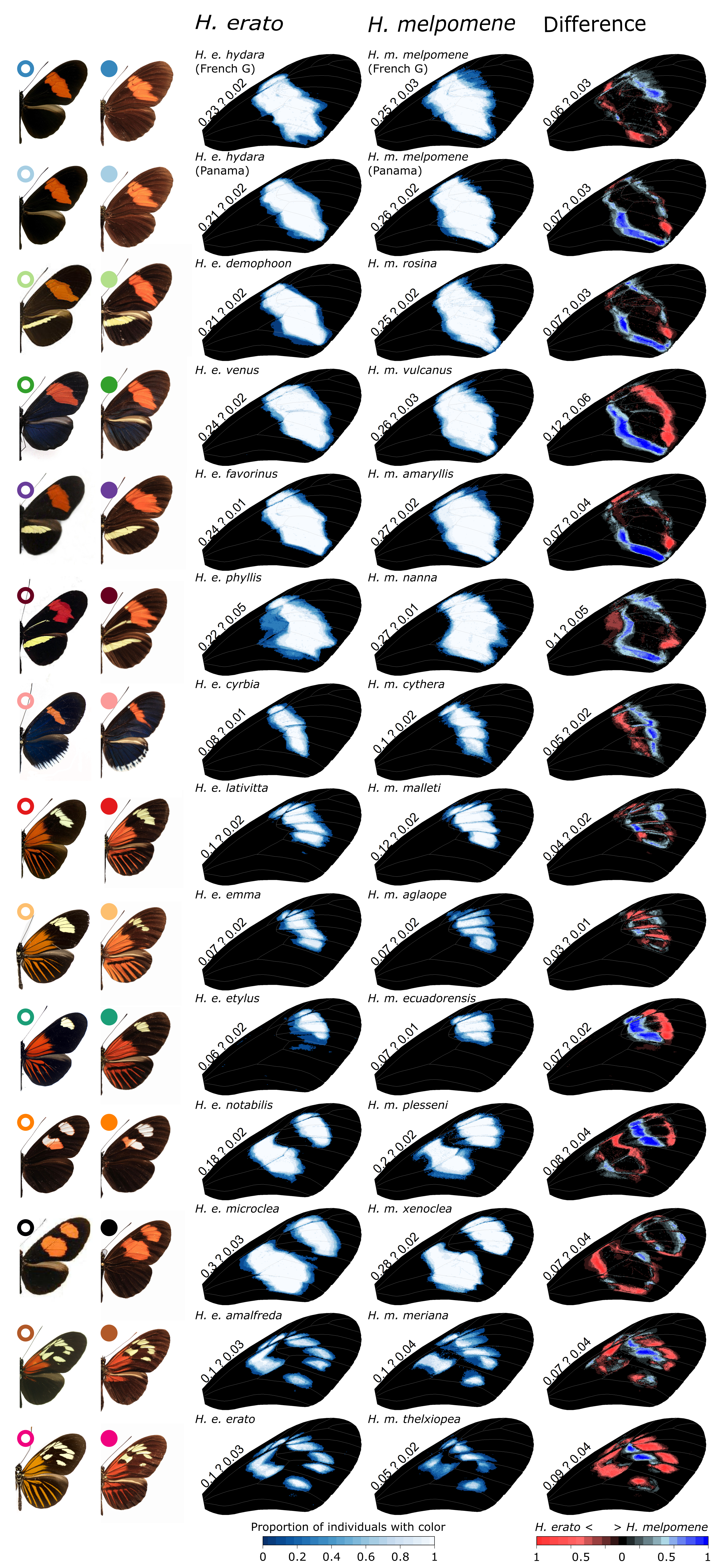

Supplement: Figure_S3.png [file rspb20201267supp3.png]

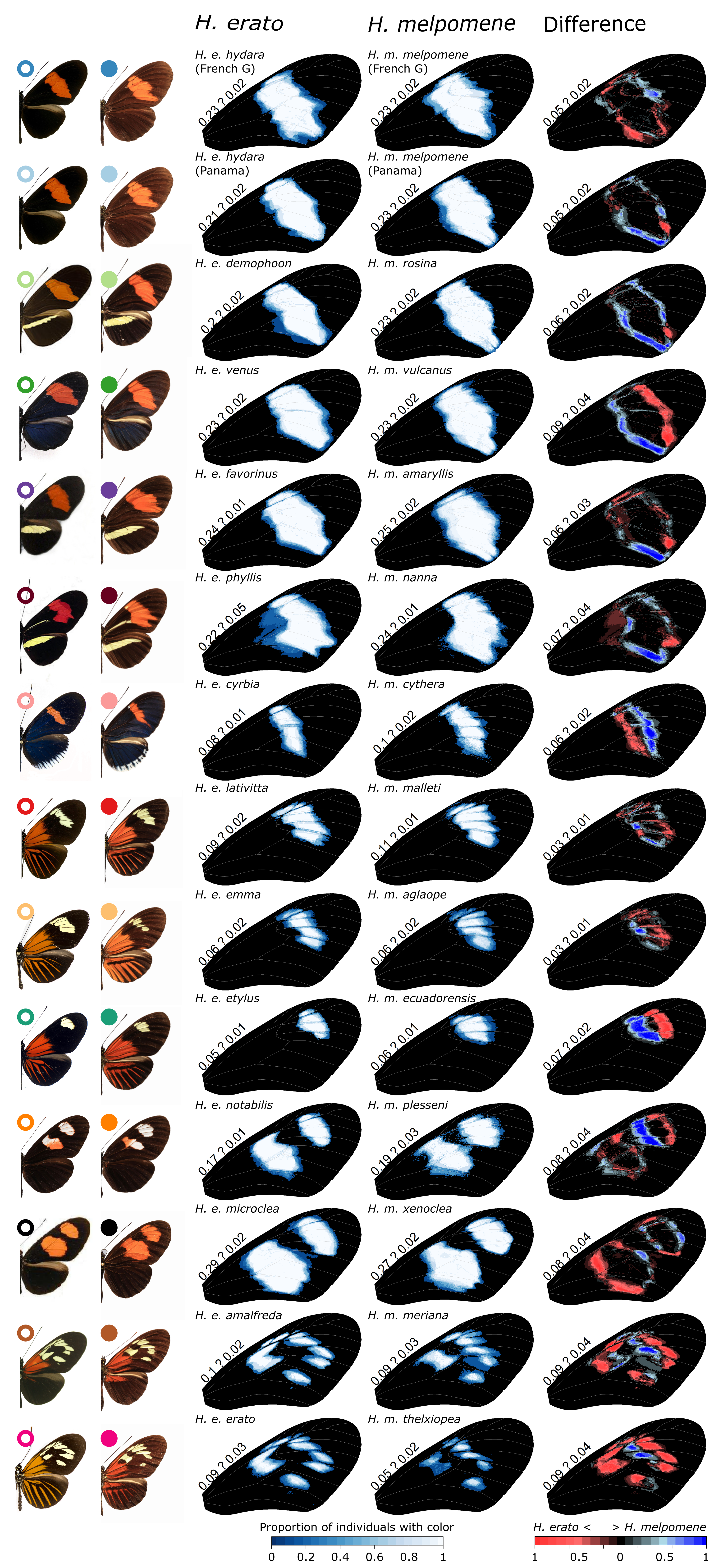

Supplement: Figure_S4.png [file rspb20201267supp4.png]
