# Supplementary material for: Perfect mimicry between Heliconius butterflies is constrained by genetics and development
Source: Proc Biol Sci. 2020 Jul 22;287(1931):20201267. doi: 10.1098/rspb.2020.1267 (PMC7423669; doi:10.1098/rspb.2020.1267)
Supplement: Table S 1. [file rspb20201267supp5.pdf]

Table S 1. Summary of sampled images of each *H. erato* and *H. melpomene* race. Symbols are as in main Figure 2.

H. erato

Race	Location	Symbol	#
H. e. hydara	Panama	○	10
H. e. hydara	French Guiana	○	10
H. e. demophoon	Panama	○	11
H. e. venus	Colombia	○	10
H. e. cyrbia	Ecuador	○	10
H. e. lativitta	Ecuador	○	10
H. e. emma	Peru	○	10
H. e. notabilis	Ecuador	○	10
H. e. etylus	Ecuador	○	10
H. e. favorinus	Peru	○	9
H. e. phyllis	Brazil	○	10
H. e. amalfreda	French Guiana	○	10
H. e. microclea	Peru	○	10
H. e. erato	Brazil, Panama, French Guiana	○	10
Total			140

H. melpomene

Race	Location	Symbol	#
H. m. melpomene	Panama	●	8
H. m. melpomene	French Guiana	●	9
H. m. rosina	Panama	●	10
H. m. vulcanus	Colombia	●	10
H. m. cythera	Ecuador	●	10
H. m. malleti	Ecuador	●	11
H. m. aglaope	Peru	●	13
H. m. plesseni	Ecuador	●	10
H. m. ecuadorensis	Ecuador	●	9
H. m. amaryllis	Peru	●	11
H. m. nanna	Brazil	●	10
H. m. meriana	French Guiana	●	8
H. m. xenoclea	Peru	●	8
H. m. thelxiopeia	French Guiana	●	14
Total			141
