# Supplementary material for: Perfect mimicry between Heliconius butterflies is constrained by genetics and development
Source: Proc Biol Sci. 2020 Jul 22;287(1931):20201267. doi: 10.1098/rspb.2020.1267 (PMC7423669; doi:10.1098/rspb.2020.1267)
Supplement: Table S 2. [file rspb20201267supp6.pdf]

Table S 2. Sample ID, sex and localities. Images can be downloaded from https://github.com/patternize-projects/Heliconius_forewing_band.

Species	Race	Image-ID	Sex	Latitude	Longitude	Location
H. erato	*amalfreda*	BC2108	m	4.941100	-55.182000	Suriname
H. erato	*amalfreda*	BC2146	m	4.958700	-55.179000	Suriname
H. erato	*amalfreda*	BC2168	f	4.943490	-55.186000	Suriname
H. erato	*amalfreda*	BC2181	m	4.940650	-55.190000	Suriname
H. erato	*amalfreda*	BC2184	f	4.940650	-55.190000	Suriname
H. erato	*amalfreda*	BC2187	m	4.938030	-55.194000	Suriname
H. erato	*amalfreda*	BC2193	f	4.932730	-55.201000	Suriname
H. erato	*amalfreda*	BC2223	f	5.261830	-55.622000	Suriname
H. erato	*amalfreda*	BC2227	m	5.234000	-55.775000	Suriname
H. erato	*amalfreda*	BC2351	f	4.983940	-55.156000	Suriname
H. erato	*cyrbia*	CAM040321	m	-0.052370	-78.766000	Ecuador
H. erato	*cyrbia*	CAM040354	m	0.207730	-78.946910	Ecuador
H. erato	*cyrbia*	CAM040355	m	0.207730	-78.946910	Ecuador
H. erato	*cyrbia*	CAM040356	m	0.207730	-78.946910	Ecuador
H. erato	*cyrbia*	CAM040357	m	0.207730	-78.946910	Ecuador
H. erato	*cyrbia*	CAM040361	f	-0.052370	-78.766000	Ecuador
H. erato	*cyrbia*	CAM040362	m	0.140570	-78.763030	Ecuador
H. erato	*cyrbia*	CAM040363	m	0.140570	-78.763030	Ecuador
H. erato	*cyrbia*	CAM040364	m	0.140570	-78.763030	Ecuador
H. erato	*cyrbia*	CAM040365	m	0.140570	-78.763030	Ecuador
H. erato	*demophoon*	IMG_1960	f	9.166333	-79.2079	Panama
H. erato	*demophoon*	IMG_1972	f	9.166333	-79.2079	Panama
H. erato	*demophoon*	IMG_1974	f	9.166333	-79.2079	Panama
H. erato	*demophoon*	IMG_1978	m	9.2074	-78.8265	Panama
H. erato	*demophoon*	IMG_1980	m	9.2074	-78.8265	Panama
H. erato	*demophoon*	IMG_1982	m	9.2074	-78.8265	Panama
H. erato	*demophoon*	IMG_1987	f	9.2074	-78.8265	Panama
H. erato	*demophoon*	IMG_2049	m	9.2481	-79.9478	Panama
H. erato	*demophoon*	IMG_2125	m	9.11605	-79.6984	Panama
H. erato	*demophoon*	IMG_2135	m	9.11605	-79.6984	Panama
H. erato	*demophoon*	IMG_2141	m	9.11605	-79.6984	Panama
H. erato	*emma*	10429077	m	-1.583333	-77.750000	Ecuador
H. erato	*emma*	10429078	m	-1.583333	-77.750000	Ecuador
H. erato	*emma*	BC2563	f	-5.29499	-78.381	Peru
H. erato	*emma*	BC2578	m	-5.29499	-78.381	Peru
H. erato	*emma*	BC2579	m	-5.29499	-78.381	Peru
H. erato	*emma*	BC2580	m	-5.29499	-78.381	Peru
H. erato	*emma*	BC2611	f	-5.38852	-78.45114	Peru
H. erato	*emma*	BC2612	m	-5.38852	-78.45114	Peru
H. erato	*emma*	BC2620	m	-5.37967	-78.45478	Peru

H. erato	*emma*	BC2624	m	-5.44466	-78.48361	Peru
H. erato	*erato*	BC0147	f	4.622068	-52.376361	French Guiana
H. erato	*erato*	BC0148	m	4.556676	-52.182277	French Guiana
H. erato	*erato*	BC0149	m	4.607970	-52.272347	French Guiana
H. erato	*erato*	BC0154	m	4.563718	-52.206697	French Guiana
H. erato	*erato*	BC0163	m	4.568734	-52.211198	French Guiana
H. erato	*erato*	BC0198	m	4.570132	-52.214164	French Guiana
H. erato	*erato*	BC0200	m	4.564127	-52.207655	French Guiana
H. erato	*erato*	BC0327	m	4.638617	-52.301596	French Guiana
H. erato	*erato*	BC0340	m	4.570132	-52.214164	French Guiana
H. erato	*erato*	BC0351	m	4.548767	-52.145497	French Guiana
H. erato	*etylus*	10429064	m	15.290278	-89.146389	Guatemala
H. erato	*etylus*	10429062	m	-1.583333	-77.750000	Ecuador
H. erato	*etylus*	BC3000	f	-2.27322	-78.19147	Ecuador
H. erato	*etylus*	BC3001	m	-2.27322	-78.19147	Ecuador
H. erato	*etylus*	BC3002	m	-2.27322	-78.19147	Ecuador
H. erato	*etylus*	BC3003	f	-2.27322	-78.19147	Ecuador
H. erato	*etylus*	BC3006	f	-2.20024	-78.15775	Ecuador
H. erato	*etylus*	BC3007	m	-2.20024	-78.15775	Ecuador
H. erato	*etylus*	BC3009	f	-2.20024	-78.15775	Ecuador
H. erato	*etylus*	BC3010	f	-2.20024	-78.15775	Ecuador
H. erato	*favorinus*	10428999	m	-9.300000	-76.000000	Peru
H. erato	*favorinus*	BC2634	m	-6.4174	-77.44329	Peru
H. erato	*favorinus*	BC2635	f	-6.4174	-77.44329	Peru
H. erato	*favorinus*	BC2636	m	-6.4174	-77.44329	Peru
H. erato	*favorinus*	BC2637	m	-6.4174	-77.44329	Peru
H. erato	*favorinus*	BC2638	m	-6.4174	-77.44329	Peru
H. erato	*favorinus*	BC2640	m	-6.4174	-77.44329	Peru
H. erato	*favorinus*	BC2643	f	-6.41388	-77.44505	Peru
H. erato	*favorinus*	BC2646	m	-6.41388	-77.44505	Peru
H. erato	*hydara*	BC0004	m	4.796800	-52.324517	French Guiana
H. erato	*hydara*	BC0049	m	4.796800	-52.324517	French Guiana
H. erato	*hydara*	BC0050	m	4.845894	-52.348475	French Guiana
H. erato	*hydara*	BC0061	f	4.549374	-52.147880	French Guiana
H. erato	*hydara*	BC0071	m	4.548767	-52.145497	French Guiana
H. erato	*hydara*	BC0076	f	4.796800	-52.324517	French Guiana
H. erato	*hydara*	BC0077	m	4.796800	-52.324517	French Guiana
H. erato	*hydara*	BC0079	m	4.796800	-52.324517	French Guiana
H. erato	*hydara*	BC0082	m	4.926844	-52.390483	French Guiana
H. erato	*hydara*	BC0125	m	4.616785	-52.283083	French Guiana
H. erato	*hydara*	IMG_1855	m	8.972917	-78.5106	Panama
H. erato	*hydara*	IMG_1861	m	8.972917	-78.5106	Panama
H. erato	*hydara*	IMG_1859	m	8.972917	-78.5106	Panama
H. erato	*hydara*	IMG_1863	f	8.950117	-78.4496	Panama

H. erato	*hydara*	IMG_1869	m	8.950117	-78.4496	Panama
H. erato	*hydara*	IMG_1871	f	8.972917	-78.5106	Panama
H. erato	*hydara*	IMG_1877	m	8.952533	-78.5251	Panama
H. erato	*hydara*	IMG_1885	f	8.972917	-78.5106	Panama
H. erato	*hydara*	IMG_1899	m	8.950117	-78.4496	Panama
H. erato	*hydara*	IMG_1993	f	9.2074	-78.8265	Panama
H. erato	*lativitta*	CAM016583	m	-1.115567	-77.778333	Ecuador
H. erato	*lativitta*	CAM016586	m	-1.115567	-77.778333	Ecuador
H. erato	*lativitta*	CAM016973	f	-1.187817	-77.831050	Ecuador
H. erato	*lativitta*	CAM016999	m	-1.187817	-77.831050	Ecuador
H. erato	*lativitta*	CAM017013	f	-1.091567	-77.719950	Ecuador
H. erato	*lativitta*	CAM017027	m	-1.091567	-77.719950	Ecuador
H. erato	*lativitta*	CAM017124	m	-1.091567	-77.719950	Ecuador
H. erato	*lativitta*	CAM017170	m	-1.061383	-77.668433	Ecuador
H. erato	*lativitta*	CAM017395	f	-1.061383	-77.668433	Ecuador
H. erato	*lativitta*	CAM017405	f	-1.098330	-77.583890	Ecuador
H. erato	*microclea*	10428928	m	-13.316667	-71.600000	Peru
H. erato	*microclea*	10428929	m	-9.883333	-74.933333	Peru
H. erato	*microclea*	10428930	m	-12.916667	-71.400000	Peru
H. erato	*microclea*	10428931	m	-11.066667	-75.316667	Peru
H. erato	*microclea*	10428932	m	-11.050000	-75.316667	Peru
H. erato	*microclea*	10428934	m	-10.883333	-75.283333	Peru
H. erato	*microclea*	10428935	m	-10.250000	-74.750000	Peru
H. erato	*microclea*	10428936	m	-10.883333	-75.216667	Peru
H. erato	*microclea*	10428937	m	-11.050000	-75.316667	Peru
H. erato	*microclea*	10428941	f	-11.066667	-75.316667	Peru
H. erato	*notabilis*	CAM016057	m	-1.383017	-77.944567	Ecuador
H. erato	*notabilis*	CAM016058	m	-1.383017	-77.944567	Ecuador
H. erato	*notabilis*	CAM016060	f	-1.383017	-77.944567	Ecuador
H. erato	*notabilis*	CAM016067	m	-1.362283	-77.959433	Ecuador
H. erato	*notabilis*	CAM016797	f	-1.429250	-77.986650	Ecuador
H. erato	*notabilis*	CAM016894	m	-1.437117	-78.122883	Ecuador
H. erato	*notabilis*	CAM016900	m	-1.437117	-78.122883	Ecuador
H. erato	*notabilis*	CAM016915	m	-1.459967	-78.072800	Ecuador
H. erato	*notabilis*	CAM017174	m	-1.367483	-78.016017	Ecuador
H. erato	*notabilis*	CAM017177	m	-1.367483	-78.016017	Ecuador
H. erato	*phyllis*	10428488	m	-16.833333	-63.916667	Bolivia
H. erato	*phyllis*	10428490	m	-17.250000	-62.750000	Bolivia
H. erato	*phyllis*	10_MARA	m	-2.614010	-44.283920	Brasil
H. erato	*phyllis*	112_BOQ2	m	-2.805583	-43.820889	Brasil
H. erato	*phyllis*	114_BOQ2	m	-2.805583	-43.820889	Brasil
H. erato	*phyllis*	35_SAG	m	-2.645028	-44.140333	Brasil
H. erato	*phyllis*	49_SAG	f	-2.645028	-44.140333	Brasil
H. erato	*phyllis*	52_SAG	m	-2.645028	-44.140333	Brasil

H. erato	*phyllis*	6_MARA	m	-2.614010	-44.283920	Brasil
H. erato	*phyllis*	85_IG	m	-2.756444	-44.321639	Brasil
H. erato	*venus*	CS000046	m	-77.373333	3.958333	Colombia
H. erato	*venus*	CS000047	f	-77.373333	3.958333	Colombia
H. erato	*venus*	CS000265	f	-77.373333	3.958333	Colombia
H. erato	*venus*	CS000275	m	-77.373333	3.958333	Colombia
H. erato	*venus*	CS000278	m	-77.373333	3.958333	Colombia
H. erato	*venus*	CS000284	m	-77.373333	3.958333	Colombia
H. erato	*venus*	CS000286	m	-77.373333	3.958333	Colombia
H. erato	*venus*	CS000288	m	-77.373333	3.958333	Colombia
H. erato	*venus*	CS003656	m	-76.757222	3.500833	Ecuador
H. erato	*venus*	CS003659	m	-76.757222	3.500833	Ecuador
H. melpomene	*agalope*	10428229	m	-4.273539	-79.204614	Ecuador
H. melpomene	*agalope*	10428230	m	-4.273539	-79.204614	Ecuador
H. melpomene	*agalope*	10428231	m	-1.583333	-77.750000	Ecuador
H. melpomene	*agalope*	10428232	m	-1.583333	-77.750000	Ecuador
H. melpomene	*agalope*	10428233	m	-1.583333	-77.750000	Ecuador
H. melpomene	*agalope*	10428234	m	-1.583333	-77.750000	Ecuador
H. melpomene	*agalope*	10428235	m	-1.583333	-77.750000	Ecuador
H. melpomene	*agalope*	10428238	f	-1.538392	-78.067322	Ecuador
H. melpomene	*agalope*	10428240	f	-1.066667	-77.550000	Ecuador
H. melpomene	*agalope*	10428266	m	-10.499805	-75.652458	Ecuador
H. melpomene	*agalope*	10428267	m	-13.433333	-70.383333	Ecuador
H. melpomene	*agalope*	CAM008689	m	-6.289700	-76.228900	Peru
H. melpomene	*agalope*	CAM008702	f	-6.239200	-76.268700	Peru
H. melpomene	*amaryllis*	10428116	m	-6.483333	-76.366667	Peru
H. melpomene	*amaryllis*	BC2639	m	-6.417400	-77.443290	Peru
H. melpomene	*amaryllis*	MJ12.3137	m	-6.454740	-76.299440	Peru
H. melpomene	*amaryllis*	MJ12.3371	m	-6.452830	-76.286215	Peru
H. melpomene	*amaryllis*	MJ12.3392	m	-6.452830	-76.286215	Peru
H. melpomene	*amaryllis*	MJ12.3393	m	-6.452830	-76.286215	Peru
H. melpomene	*amaryllis*	MJ12.3396	m	-6.452830	-76.286215	Peru
H. melpomene	*amaryllis*	MJ12.3414	m	-6.454010	-76.300230	Peru
H. melpomene	*amaryllis*	MJ12.3416	m	-6.453700	-76.298070	Peru
H. melpomene	*amaryllis*	MJ12.3417	m	-6.453700	-76.298070	Peru
H. melpomene	*amaryllis*	MJ12.3442	m	-6.454740	-76.299440	Peru
H. melpomene	*cythera*	15N005	f	0.175330	-78.907520	Ecuador
H. melpomene	*cythera*	15N006	m	0.193660	-78.858700	Ecuador
H. melpomene	*cythera*	15N009	m	0.175330	-78.907520	Ecuador
H. melpomene	*cythera*	15N020	m	0.184970	-78.853020	Ecuador
H. melpomene	*cythera*	15N022	f	0.184970	-78.853020	Ecuador
H. melpomene	*cythera*	15N023	m	0.184970	-78.853020	Ecuador
H. melpomene	*cythera*	CAM008510	m	-0.648000	-78.789500	Ecuador
H. melpomene	*cythera*	CAM040383	f	0.212420	-78.938550	Ecuador

H. melpomene	*cythera*	CAM040459	m	0.149100	-78.759070	Ecuador
H. melpomene	*cythera*	CAM040474	f	0.153760	-78.758970	Ecuador
H. melpomene	*ecuadorensis*	10428219	m	-4.066667	-78.966667	Ecuador
H. melpomene	*ecuadorensis*	10428220	m	-1.583333	-77.750000	Ecuador
H. melpomene	*ecuadorensis*	CAM009112	m	-4.043900	-78.986100	Ecuador
H. melpomene	*ecuadorensis*	CAM009113	m	-4.043900	-78.986100	Ecuador
H. melpomene	*ecuadorensis*	CAM009114	m	-4.043900	-78.986100	Ecuador
H. melpomene	*ecuadorensis*	CAM009115	m	-4.043900	-78.986100	Ecuador
H. melpomene	*ecuadorensis*	CAM009119	m	-4.043900	-78.986100	Ecuador
H. melpomene	*ecuadorensis*	CAM009120	m	-4.043900	-78.986100	Ecuador
H. melpomene	*ecuadorensis*	CAM009141	m	-2.251000	-78.200000	Ecuador
H. melpomene	*malleti*	CAM016772	m	-1.251850	-77.819600	Ecuador
H. melpomene	*malleti*	CAM016224	f	-1.115567	-77.778333	Ecuador
H. melpomene	*malleti*	CAM016606	m	-1.115567	-77.778333	Ecuador
H. melpomene	*malleti*	CAM016144	m	-1.416050	-77.729017	Ecuador
H. melpomene	*malleti*	CAM016267	m	-1.250967	-77.698850	Ecuador
H. melpomene	*malleti*	CAM016609	f	-1.115567	-77.778333	Ecuador
H. melpomene	*malleti*	CAM016610	m	-1.115567	-77.778333	Ecuador
H. melpomene	*malleti*	CAM016611	m	-1.115567	-77.778333	Ecuador
H. melpomene	*malleti*	CAM017049	m	-1.168350	-77.781117	Ecuador
H. melpomene	*malleti*	CAM017064	m	-1.168350	-77.781117	Ecuador
H. melpomene	*malleti*	CAM017070	m	-1.168350	-77.781117	Ecuador
H. melpomene	*melpomene*	10428369	f	5.500000	-54.033300	French Guiana
H. melpomene	*melpomene*	10428371	f	5.400000	-54.083300	French Guiana
H. melpomene	*melpomene*	CAM000413	m	4.913800	-52.359500	French Guiana
H. melpomene	*melpomene*	CAM001422	m	4.963200	-52.420000	French Guiana
H. melpomene	*melpomene*	CAM008171	f	4.963200	-52.420000	French Guiana
H. melpomene	*melpomene*	CAM008215	m	4.789000	-52.404000	French Guiana
H. melpomene	*melpomene*	CAM008218	m	4.963200	-52.420000	French Guiana
H. melpomene	*melpomene*	CAM008863	m	7.756800	-77.684100	Panama
H. melpomene	*melpomene*	CAM008887	m	7.756800	-77.684100	Panama
H. melpomene	*melpomene*	CAM008888	m	7.756800	-77.684100	Panama
H. melpomene	*melpomene*	CAM008954	m	7.636200	-78.189700	Panama
H. melpomene	*melpomene*	CAM008955	m	7.636200	-78.189700	Panama
H. melpomene	*melpomene*	CAM008956	m	7.636200	-78.189700	Panama
H. melpomene	*melpomene*	CAM008957	m	7.636200	-78.189700	Panama
H. melpomene	*melpomene*	CAM008979	m	7.636200	-78.189700	Panama
H. melpomene	*melpomene*	CAM009316	m	4.963200	-52.420000	French Guiana
H. melpomene	*melpomene*	CAM009317	m	4.963200	-52.420000	French Guiana
H. melpomene	*meriana*	13715	m	3.688300	-54.082500	Suriname
H. melpomene	*meriana*	melp_14-103	m	5.113892	-54.990106	Suriname
H. melpomene	*meriana*	melp_14-108	m	5.113892	-54.990106	Suriname
H. melpomene	*meriana*	melp_14-110	m	5.113892	-54.990106	Suriname
H. melpomene	*meriana*	melp_14-111	m	5.113892	-54.990106	Suriname

H. melpomene	*meriana*	melp_14-122	m	5.113892	-54.990106	Suriname
H. melpomene	*meriana*	melp_14-133	f	5.113892	-54.990106	Suriname
H. melpomene	*meriana*	melp_14-138	f	5.113892	-54.990106	Suriname
H. melpomene	*nanna*	LMCI_105-62	m	-19.059167	-40.139722	Brasil
H. melpomene	*nanna*	LMCI_105-63	m	-19.059167	-40.139722	Brasil
H. melpomene	*nanna*	LMCI_105-64	f	-19.059167	-40.139722	Brasil
H. melpomene	*nanna*	LMCI_183-10	m	-3.754444	-40.910278	Brasil
H. melpomene	*nanna*	LMCI_183-11	m	-3.754444	-40.910278	Brasil
H. melpomene	*nanna*	LMCI_183-14	m	-3.754444	-40.910278	Brasil
H. melpomene	*nanna*	LMCI_183-15	m	-3.754444	-40.910278	Brasil
H. melpomene	*nanna*	LMCI_183-16	m	-3.754444	-40.910278	Brasil
H. melpomene	*nanna*	LMCI_183-18	f	-3.754444	-40.910278	Brasil
H. melpomene	*nanna*	LMCI_183-19	m	-3.754444	-40.910278	Brasil
H. melpomene	*plesseni*	CAM016347	f	-1.425867	-78.018200	Ecuador
H. melpomene	*plesseni*	CAM016349	f	-1.425867	-78.018200	Ecuador
H. melpomene	*plesseni*	CAM016354	m	-1.425867	-78.018200	Ecuador
H. melpomene	*plesseni*	CAM016355	m	-1.425867	-78.018200	Ecuador
H. melpomene	*plesseni*	CAM016378	m	-1.425867	-78.018200	Ecuador
H. melpomene	*plesseni*	CAM016810	f	-1.409933	-77.915350	Ecuador
H. melpomene	*plesseni*	CAM017185	m	-1.367483	-78.016017	Ecuador
H. melpomene	*plesseni*	CAM017186	f	-1.367483	-78.016017	Ecuador
H. melpomene	*plesseni*	CAM017187	f	-1.367483	-78.016017	Ecuador
H. melpomene	*plesseni*	CAM017614	m	-1.422417	-78.172933	Ecuador
H. melpomene	*rosina*	CAM000903	f	9.129900	-79.715800	Panama
H. melpomene	*rosina*	CAM000947	m	9.129900	-79.715800	Panama
H. melpomene	*rosina*	CAM001015	m	9.116000	-79.698000	Panama
H. melpomene	*rosina*	CAM001027	m	9.076000	-79.659000	Panama
H. melpomene	*rosina*	CAM001067	m	9.010900	-79.547700	Panama
H. melpomene	*rosina*	CAM001137	f	9.122200	-79.714900	Panama
H. melpomene	*rosina*	CAM001391	f	9.122200	-79.714900	Panama
H. melpomene	*rosina*	CAM002901	m	NA	NA	Panama
H. melpomene	*rosina*	CAM008052	m	8.981000	-82.240000	Panama
H. melpomene	*rosina*	CAM009554	m	8.975800	-78.375300	Panama
H. melpomene	*thelxiopea*	10428329	m	5.250000	-54.250000	French Guiana
H. melpomene	*thelxiopea*	10428331	f	5.400000	-54.083333	French Guiana
H. melpomene	*thelxiopea*	10428332	f	5.400000	-54.083333	French Guiana
H. melpomene	*thelxiopea*	10428333	m	5.400000	-54.083333	French Guiana
H. melpomene	*thelxiopea*	10428334	m	5.400000	-54.083333	French Guiana
H. melpomene	*thelxiopea*	10428335	f	5.400000	-54.083333	French Guiana
H. melpomene	*thelxiopea*	10428336	f	5.400000	-54.083333	French Guiana
H. melpomene	*thelxiopea*	10428337	m	5.400000	-54.083333	French Guiana
H. melpomene	*thelxiopea*	10428338	m	5.250000	-54.250000	French Guiana
H. melpomene	*thelxiopea*	10428339	m	5.500000	-54.033333	French Guiana
H. melpomene	*thelxiopea*	10428340	f	5.400000	-54.083333	French Guiana

H. melpomene	*thelxiopea*	10428341	f	5.250000	-54.250000	French Guiana
H. melpomene	*thelxiopea*	10428342	f	5.516667	-53.966667	French Guiana
H. melpomene	*thelxiopea*	10428343	m	5.500000	-54.033333	French Guiana
H. melpomene	*vulcanus*	CAM000058	m	3.777900	-76.130000	Colombia
H. melpomene	*vulcanus*	CAM000059	m	3.782500	-76.721100	Colombia
H. melpomene	*vulcanus*	CAM000060	m	3.782500	-76.721100	Colombia
H. melpomene	*vulcanus*	CAM000061	m	3.782500	-76.721100	Colombia
H. melpomene	*vulcanus*	CAM000062	m	3.782500	-76.721100	Colombia
H. melpomene	*vulcanus*	CAM000063	m	3.782500	-76.721100	Colombia
H. melpomene	*vulcanus*	CAM000064	m	3.782500	-76.721100	Colombia
H. melpomene	*vulcanus*	CAM000129	m	3.895800	-76.621400	Colombia
H. melpomene	*vulcanus*	CAM000132	m	3.895800	-76.621400	Colombia
H. melpomene	*vulcanus*	CAM000134	m	3.895800	-76.621400	Colombia
H. melpomene	*xenoclea*	MJ12.3605	m	-11.174530	-75.403470	Peru
H. melpomene	*xenoclea*	MJ12.3606	m	-11.174530	-75.403470	Peru
H. melpomene	*xenoclea*	MJ12.3608	m	-11.174530	-75.403470	Peru
H. melpomene	*xenoclea*	MJ12.3636	m	-11.033770	-75.409130	Peru
H. melpomene	*xenoclea*	MJ12.3638	m	-11.036420	-75.407990	Peru
H. melpomene	*xenoclea*	MJ12.3647	m	-11.044580	-75.413270	Peru
H. melpomene	*xenoclea*	MJ12.3648	m	-11.033770	-75.409130	Peru
H. melpomene	*xenoclea*	MJ12.3651	m	-11.033770	-75.409130	Peru
H. melpomene	*xenoclea*	MJ12.3653	m	-11.033770	-75.409130	Peru
