# Supplementary material for: Perfect mimicry between Heliconius butterflies is constrained by genetics and development
Source: Proc Biol Sci. 2020 Jul 22;287(1931):20201267. doi: 10.1098/rspb.2020.1267 (PMC7423669; doi:10.1098/rspb.2020.1267)
Supplement: Table S 3. [file rspb20201267supp7.pdf]

Table S 3. MANOVA and LDA results for shape variation in the forewing of *H. erato* and *H. melpomene*. (A) MANOVA and LDA results including all 18 landmarks. **(B)** MANOVA and LDA results including subset of landmarks. Only the scores of samples along significant PC axes were used as determined by permutation.

A. All 18 landmarks					
Permutation (Jackstraw): N significant PCs					6.00
	Pillai	F	DF	p	
Manova PC 1-6 ~ species	0.66	86.60	1,280	< 2.2e-16	
Manova PC 1-6 ~ sex	0.24	14.41	1,280	2.86E-14	
Manova PC 1-6 ~ species*sex	0.04	1.68	1,280	0.1271	
Manova each PC ~ species	Pillai	F	DF	p	variance explained
PC1		84.96	1,280	7.91E-18	0.23
PC2		134.95	1,280	1.11E-25	0.17
PC3		12.40	1,280	5.03E-04	0.09
PC4		3.53	1,280	6.14E-02	0.07
PC5		5.31	1,280	2.19E-02	0.07
PC6		15.69	1,280	9.51E-05	0.06
Manova each PC ~ sex	Pillai	F	DF	p	variance explained
PC1		33.06	1,280	2.36E-08	0.23
PC2		18.18	1,280	2.76E-05	0.17
PC3		0.67	1,280	4.15E-01	0.09
PC4		0.48	1,280	4.87E-01	0.07
PC5		30.59	1,280	7.37E-08	0.07
PC6		1.57	1,280	2.11E-01	0.06

LDA classification ~ species	
Species	posterior
H. erato	94.29
H. melpomene	87.23

LDA classification ~ sex	
Sex	posterior
male	74.65
female	67.65

B. Subset landmarks					
Permutation (Jackstraw): N significant PCs					5.00
	Pillai	F	DF	p	
Manova PC 1-6 ~ species	0.22	15.49	1,280	2.07E-13	
Manova PC 1-6 ~ sex	0.20	13.95	1,280	3.77E-12	
Manova PC 1-6 ~ species*sex	0.02	1.36	1,280	0.238	
Manova each PC ~ species	Pillai	F	DF	p	variance explained
PC1		9.32	1,280	2.49E-03	0.23
PC2		45.03	1,280	1.09E-10	0.19
PC3		9.46	1,280	2.32E-03	0.15
PC4		2.36	1,280	1.26E-01	0.10
PC5		0.02	1,280	9.01E-01	0.08
Manova each PC ~ sex	Pillai	F	DF	p	variance explained
PC1		45.25	1,280	9.90E-11	0.23
PC2		0.00	1,280	9.53E-01	0.19
PC3		12.12	1,280	5.80E-04	0.15
PC4		0.48	1,280	4.90E-01	0.10
PC5		3.33	1,280	6.91E-02	0.08

LDA classification ~ species	
Species	posterior
H. erato	61.43
H. melpomene	68.09

LDA classification ~ sex	
Sex	posterior
male	67.61
female	63.24
