# Supplementary material for: Perfect mimicry between Heliconius butterflies is constrained by genetics and development
Source: Proc Biol Sci. 2020 Jul 22;287(1931):20201267. doi: 10.1098/rspb.2020.1267 (PMC7423669; doi:10.1098/rspb.2020.1267)
Supplement: Table S 4. [file rspb20201267supp8.pdf]

Table S 4. MANOVA and LDA results for mid-forewing band (MFB) shape variation in *H. erato* and *H. melpomene* using the subset of landmarks. (A) MANOVA and LDA for *H. erato*. **(B)** MANOVA and LDA for *H. melpomene*. **(C)** MANOVA and LDA for *H. erato* and *H. melpomene* combined. **(D)** MANOVA and LDA for *H. erato* and *H. melpomene* Postman races combined. Only the scores of samples along significant PC axes were used as determined by permutation.

A. Subset landmarks	PCA *H. erato*			
	Permutation (Jackstraw): N significant PCs			10.00
	F	DF	p	
Manova PC 1-10 ~ population	35.39	13,139	< 2.2e-16	
Manova PC 1-10 ~ sex	3.11	1,139	0.00168	
Manova PC 1-10 ~ population*sex	1.24	13,139	0.04399	
Manova each PC ~ population	F	DF	p	variance explained
PC1	575.39	13,139	5.14E-96	0.33
PC2	172.97	13,139	1.10E-67	0.20
PC3	60.12	13,139	2.95E-44	0.07
PC4	121.26	13,139	1.40E-59	0.05
PC5	98.84	13,139	5.26E-55	0.03
PC6	40.07	13,139	5.17E-36	0.03
PC7	56.97	13,139	3.94E-43	0.02
PC8	28.40	13,139	1.65E-29	0.01
PC9	7.56	13,139	1.57E-10	0.01
PC10	8.33	13,139	1.54E-11	0.01
Manova each PC ~ sex	F	DF	p	variance explained
PC1	1.39	1,139	2.41E-01	0.33
PC2	0.03	1,139	8.52E-01	0.20
PC3	2.01	1,139	1.59E-01	0.07
PC4	0.58	1,139	4.49E-01	0.05
PC5	3.17	1,139	7.76E-02	0.03
PC6	7.50	1,139	7.19E-03	0.03
PC7	1.99	1,139	1.61E-01	0.02
PC8	9.98	1,139	2.04E-03	0.01
PC9	1.46	1,139	2.30E-01	0.01
PC10	9.03	1,139	3.28E-03	0.01

LDA classification ~ populations	
Population	posterior
H. e. hydara (Panama)	60
H. e. hydara (French Guiana)	90
H. e. demophoon	54.55
H. e. venus	90
H. e. cyrbia	100
H. e. lativitta	80
H. e. emma	90
H. e. notabilis	100
H. e. etylus	100
H. e. favorinus	66.67
H. e. phyllis	90
H. e. amalfreda	60
H. e. erato	60
H. e. microclea	100

LDA classification ~ populations	
Sex	posterior
male	55.77
female	58.33

B. Subset landmarks		PCA *H. melpomene*		
Permutation (Jackstraw): N significant PCs				11.00
	F	DF	p	
Manova PC 1-11 ~ population	19.73	13,140	< 2.2e-16	
Manova PC 1-11 ~ sex	3.17	1,140	0.00103	
Manova PC 1-11 ~ population*sex	1.27	8,140	0.05324	
Manova each PC ~ population	F	DF	p	variance explained
PC1	603.97	13,140	8.67E-96	0.36
PC2	238.15	13,140	4.13E-74	0.18
PC3	44.28	13,140	1.35E-37	0.08
PC4	56.07	13,140	2.47E-42	0.04
PC5	35.73	13,140	1.85E-33	0.02
PC6	45.37	13,140	4.48E-38	0.02
PC7	15.15	13,140	5.07E-19	0.02
PC8	8.43	13,140	1.33E-11	0.01
PC9	13.28	13,140	3.61E-17	0.01
PC10	8.22	13,140	2.48E-11	0.01
PC11	1.36	13,140	1.92E-01	0.01
Manova each PC ~ sex	F	DF	p	variance explained
PC1	0.84	1,140	3.61E-01	0.36
PC2	0.00	1,140	9.56E-01	0.18
PC3	1.59	1,140	2.10E-01	0.08
PC4	0.77	1,140	3.81E-01	0.04
PC5	0.02	1,140	8.94E-01	0.02
PC6	4.88	1,140	2.91E-02	0.02
PC7	2.13	1,140	1.47E-01	0.02
PC8	1.69	1,140	1.96E-01	0.01
PC9	2.10	1,140	1.50E-01	0.01
PC10	20.92	1,140	1.19E-05	0.01
PC11	6.05	1,140	1.54E-02	0.01

LDA classification ~ populations	
Population	posterior
H. m. melpomene (Panama)	25
H. m. melpomene (F. Guiana)	88.89
H. m. rosina	40
H. m. vulcanus	10
H. m. cythera	100
H. m. malleti	90.91
H. m. aglaope	76.92
H. m. plesseni	100
H. m. ecuadorensis	100
H. m. amaryllis	63.64
H. m. nanna	100
H. m. meriana	75
H. m. thelxiopeia	85.71
H. m. xenoclea	100

LDA classification ~ populations	
Sex	posterior
male	77.98
female	62.5

C. Subset landmarks			PCA H. erato + H. melpomene	
Permutation (Jackstraw): N significant PCs				18.00
	F	DF	p	
Manova PC 1-18 ~ population	18.01	26,280	< 2.2e-16	
Manova PC 1-18 ~ species	70.77	1,280	< 2.2e-16	
Manova each PC ~species	F	DF	p	variance explained
PC1	61.57	1,280	1.21E-13	0.32
PC2	73.32	1,280	1.09E-15	0.19
PC3	1.74	1,280	1.88E-01	0.07
PC4	0.22	1,280	6.42E-01	0.04
PC5	300.78	1,280	6.21E-45	0.03
PC6	218.69	1,280	4.38E-36	0.03
PC7	14.78	1,280	1.53E-04	0.02
PC8	84.12	1,280	1.68E-17	0.02
PC9	141.21	1,280	3.59E-26	0.01
PC10	29.54	1,280	1.29E-07	0.01
PC11	10.30	1,280	1.50E-03	0.01
PC12	11.28	1,280	9.04E-04	0.01
PC13	9.78	1,280	1.97E-03	0.01
PC14	30.50	1,280	8.28E-08	0.01
PC15	0.10	1,280	7.58E-01	0.01
PC16	0.37	1,280	5.43E-01	0.01
PC17	0.19	1,280	6.64E-01	0.00
PC18	0.16	1,280	6.94E-01	0.00

LDA classification ~ species	
Species	posterior
H. erato	88.57
H. melpomene	92.2

D. Subset landmarks			PCA H. erato + H. melpomene Postman	
Permutation (Jackstraw): N significant PCs				15.00
	F	DF	p	
Manova PC 1-18 ~ population	6.25	10,127	< 2.2e-16	
Manova PC 1-18 ~ species	70.02	1,127	< 2.2e-16	
Manova each PC ~species	F	DF	p	variance explained
PC1	14.55	1,127	2.30E-04	0.26
PC2	131.01	1,127	3.11E-20	0.10
PC3	184.05	1,127	6.53E-25	0.08
PC4	23.81	1,127	3.77E-06	0.05
PC5	0.01	1,127	9.37E-01	0.03
PC6	5.81	1,127	1.77E-02	0.02
PC7	0.03	1,127	8.67E-01	0.02
PC8	2.56	1,127	1.13E-01	0.02
PC9	0.49	1,127	4.86E-01	0.02
PC10	2.22	1,127	1.39E-01	0.02
PC11	0.44	1,127	5.09E-01	0.01
PC12	0.69	1,127	4.10E-01	0.01
PC13	1.93	1,127	1.67E-01	0.01
PC14	1.31	1,127	2.54E-01	0.01
PC15	0.32	1,127	5.72E-01	0.01

LDA classification ~ species	
Species	posterior
H. erato	100
H. melpomene	98.3
