# Supplementary material for: Perfect mimicry between Heliconius butterflies is constrained by genetics and development
Source: Proc Biol Sci. 2020 Jul 22;287(1931):20201267. doi: 10.1098/rspb.2020.1267 (PMC7423669; doi:10.1098/rspb.2020.1267)
Supplement: Supplementary material S1 [file rspb20201267supp9.pdf]

Proceedings of the Royal Society B

Perfect mimicry between *Heliconius* butterflies is constrained by genetics and development

Steven M. Van Belleghem[1,*], Paola A. Alicea Roman[1,2], Heriberto Carbia Gutierrez[1], Brian A. Counterman[3] and Riccardo Papa[1]

[1]Department of Biology, University of Puerto Rico, Rio Piedras, Puerto Rico.

[2] Department of Biology, University of Puerto Rico, Humacao, Puerto Rico.

[3]Department of Biological Sciences, Mississippi State University, Mississippi State, USA.

[*] VanBelleghemSteven@hotmail.com

Supplementary materials S1 - *interspecific changes in wing shape and sex differences*

Methods

Interspecific wing shape differences, as previously identified between *H. erato* and *H. melpomene* [1,2], could potentially affect color pattern comparisons by overcompensating the pattern alignment compared to its relative position in the wing. Therefore, we compared two sets of landmarks: (1) one with all 18 vein intersection points and (2) a second analysis excluding landmarks that caused interspecific tension in the alignment (Figure S1). Landmarks were placed on one forewing of each individual using *ImageJ* [3]. Landmarks were superimposed using Procrustes superimposition with the *procSym* function in the R package *Morpho* [4]. This superimposition transforms the raw landmark coordinates to a common centroid, scaling to unit centroid size, and rotating the shapes until the sum of squared distances between landmarks is minimized. The resulting Procrustes coordinates then describe shape differences between the samples. Tension maps (Figure S1) represent the Euclidean distance between the average *H. erato* and *H. melpomene* Procrustes landmark arrangement and were created with a modified *tps_iso* and *tps_arr* function of the R package *Momocs* [5]. Landmark Principal Component Analysis (PCA) was performed with the *procSym* function in the R package *Morpho* [4] and ignoring size differences between wings (i.e. *sizeshape = FALSE*).

To test the effect of sex and species on shape variables, we used a two-way multivariate analysis of variance (MANOVA) as implemented in R v3.5.1 [6]. For this, we used only the values of samples along significant PC axes as determined by the *permutationPA* function in the R package *jackstraw* [7]. Shape

discrimination between *H. erato* and *H. melpomene* and posterior probability of classification was studied using linear discriminant analysis (LDA) as implemented in the R package *MASS* [8].

Results

We investigated potential non-linear interspecific differences in wing shape that might affect the downstream color pattern analysis. PCA of the complete set of 18 landmarks placed at the intersection of wing veins in the total set of 281 samples showed significant interspecific wing shape differences with a posterior probability of classification of 94.3 % and 87.2 % for *H. erato* and *H. melpomene*, respectively ($F_{1,280}$ = 86.6, $p < 0.001$; Table S3; Figure S1A, B). These differences were mainly concentrated in the first two PC axes which explained a combined 40 % of the variation in the dataset (Table S3). Interspecific shape differences were most apparent at landmarks 2, 3, 7, 8 and 9 and affected mostly the alignment at the posterior proximal to medial area and the anterior medial to distal area of the wing (red areas in top part Figure S1B). While significant interspecific differences remained after occluding landmarks 2-5 and 7-9 ($F_{1,280}$ = 15.5, $p < 0.001$; Table S3), the posterior probability of classification dropped to 61.4 % and 68.1 % for *H. erato* and *H. melpomene*, respectively (Table S3). Only small interspecific tension areas remained in the alignment in the subset landmark alignment (Figure S1B). Therefore, in the next sections describing the MFB color pattern analysis we mainly present results obtained from the subset landmark set.

Comparing wing shape between males and females using the subset landmark set showed significant differences in sex in both *H. erato* and *H. melpomene* mostly along the first PC axis ($F_{1,280}$ = 14.0, $p < 0.001$; Table S3; Figure S1C). However, sex had generally a low posterior probability of classification of 67.6 % and 63.2 % for males and females, respectively, indicating large overlap in the phenotypes of the sex classes.

Despite existing interspecific shape changes observed between *H. erato* and *H. melpomene* (Supplementary Materials S1; Table S4; Figure S1), wing color pattern alignments using an extended set of 18 landmarks showed very similar PCA clustering for all populations and phenotypes compared to the subset of 11 landmark analysis (Figure S2, 3). Similarly, removing females from our dataset did not change the results (Figure S4).

References

1. Mérot C, Le Poul Y, Théry M, Joron M. 2016 Refining mimicry: phenotypic variation tracks the local optimum. *J. Anim. Ecol.* **85**, 1056–1069.
2. Rossato DO, Boligon D, Fornel R, Kronforst MR, Gonçalves GL, Moreira GRP. 2018 Subtle variation in size and shape of the whole forewing and the red band among co-mimics revealed by geometric morphometric analysis in *Heliconius* butterflies. *Ecol. Evol.* **8**, 3280–3295.
3. Schindelin J, Rueden CT, Hiner MC, Eliceiri KW. 2015 The ImageJ ecosystem: An open platform for biomedical image analysis. *Mol. Reprod. Dev.* **82**, 518–529.
4. Schlager S. 2016 Morpho: Calculations and visualisations related to geometric morphometrics. R package version 2.3.1.1. http://cran.r-project.org/package=Morpho.
5. Bonhomme V, Picq S, Gaucherel C, Claude J. 2014 Momocs: Outline analysis using R. *J. Stat. Softw.* **56**, 1–24.
6. R Core Team. 2018 R: A language and environment for statistical computing.
7. Chung NC, Storey JD. 2015 Statistical significance of variables driving systematic variation in high-dimensional data. *Bioinformatics* **31**, 545–554.
8. Ripley B, Bates D, Hornik K, Gebhardt A, Firth D. 2019 Package 'MASS'.
